# Food-Borne *Vibrio parahaemolyticus* in China: Prevalence, Antibiotic Susceptibility, and Genetic Characterization

**DOI:** 10.3389/fmicb.2020.01670

**Published:** 2020-07-16

**Authors:** Yanping Li, Tengfei Xie, Rui Pang, Qingping Wu, Jumei Zhang, Tao Lei, Liang Xue, Haoming Wu, Juan Wang, Yu Ding, Moutong Chen, Shi Wu, Haiyan Zeng, Youxiong Zhang, Xianhu Wei

**Affiliations:** ^1^College of Food Science, South China Agricultural University, Guangzhou, China; ^2^Guangdong Institute of Microbiology, State Key Laboratory of Applied Microbiology Southern China, Guangdong Provincial Key Laboratory of Microbial Safety and Health, Guangdong Academy of Sciences, Guangzhou, China; ^3^Department of Food Science and Technology, Jinan University, Guangzhou, China

**Keywords:** *Vibrio parahaemolyticus*, prevalent, antibiotic resistance, serogroup, virulence gene, MLST

## Abstract

*Vibrio parahaemolyticus* is a marine and estuarine bacterium that leads to damage of aquatic industry by foodborne outbreaks and possesses an enormous threat to food safety as well as human health worldwide. In the current study, we investigated 905 food samples (ready-to-eat foods, fish, and shrimp) from 15 provinces in China, and aimed to determine prevalence, biological characteristics and genetic diversity of presumptive *V. parahaemolyticus* isolates. Firstly, 14.17% of 240 fish samples, 15.34% of 365 shrimp samples and 3.67% of 300 RTE food samples were positive for potential *V. parahaemolyticus*. Secondly, 69 food samples (14.87%) collected in summer were positive for target isolates, while the rate of positive sample of 441 food samples in winter reached 7.26%. Thirdly, we purified 202 *V. parahaemolyticus* strains for further research. And antimicrobial susceptibility results of strains tested revealed that the highest resistance rate was observed for ampicillin (79.20%). At the same time, 148 (73.27%) of all isolates were classified and defined as multi-drug resistant foodborne bacteria. The results of PCR assay showed that the isolates being positive for the *tdh*, *trh* or both genes, were up to 9.90%, 19.80% or 3.96%. Besides, multiplex PCR test showed that the isolates carrying O2 serogroup were the most prevalent. Furthermore, sequence types (STs) of 108 isolates were obtained via multi-locus sequence typing. Not only 82 STs were detected, but also 41 of which were updated in the MLST database. Thus, our findings significantly demonstrated the high contamination rates of *V. parahaemolyticus* in fish and shrimp and it may possess potential threat for consumer health. We also provided up-to-date dissemination of antibiotic-resistant *V. parahaemolyticus* which is important to ensure the high efficacy in the treatment of human and aquatic products infections. Lastly, with the identification of 82 STs including 41 novel STs, this study significantly revealed the high genetic diversity among *V. parahaemolyticus*. All of our research improved our understanding on microbiological risk assessment in ready-to-eat foods, fish, and shrimp.

## Introduction

*Vibrio parahaemolyticus*, a food-borne gastroenteritis-causing bacterium, is identified as a gram-negative bacterial cell that is common in seawater, seafood, and aquatic products. *V. parahaemolyticus* gastroenteritis outbreaks have been reported worldwide, including those in Bangladesh (Akther et al., 2016), Europe (Baker-Austin et al., 2010; Caburlotto et al., 2016; Lopatek et al., 2018), Japan (Arakawa et al., 1999), United States (Shaw et al., 2015) and South America (Raszl et al., 2016). In China, this bacterium is also a common causative agent of food poisoning (Xie et al., 2015; Dong et al., 2016) associated with consumption of fish, shellfish, and shrimp. Recently, not only has it been isolated from samples of a variety of aquatic products, but it has also been found in ready-to-eat (RTE) food (Xie et al., 2015; Cho et al., 2016; Pang et al., 2019). However, to date, the limited risk assessment of *V. parahaemolyticus* on its prevalence and contamination levels of RTE food leaded less information about monitoring and treatment strategies in China. Therefore, we presented information and provided insights indicating the importance of developing microbiological risk assessment and control strategies for *V. parahaemolyticus* strains closely related to food safety into the future.

Since the discovery of penicillin in the 1920s, the application of antibiotics makes great contribution to human and animal medical treatment (Aarestrup and Wegener, 1999). Nowadays, increasing researches have obviously indicated that prevalence of antibiotic-resistant *V. parahaemolyticus* may pose a huge threat to public health and economic development for humans worldwide (Ghenem and Elhadi, 2018; Lopatek et al., 2018). During the past few decades, due to the excessive application of antibiotics in medical treatment and aquaculture industry, antibiotic resistance has emerged and evolved in *V. parahaemolyticus* (Mazel and Davies, 1999; Cabello, 2006; Elmahdi et al., 2016). Currently, a large number of reports indicate that *V. parahaemolyticus* isolated from various sources has presented high resistance to single or multiple antibiotics, especially ampicillin (Lee et al., 2018; Fattel et al., 2019; Jiang et al., 2019; Mohamad et al., 2019; Ryu et al., 2019). The frequently occurring phenomenon of multi-drug resistance in *V. parahaemolyticus* directly affects application of antibiotics and prevention as well as treatment of bacterial infectious diseases (Xu et al., 2016). Therefore, it is urgent and necessary to establish a monitoring system and efficient treatment strategies of the *V. parahaemolyticus* antimicrobial-resistance profile.

Food poisoning associated with pathogenic *V. parahaemolyticus* is often caused by consumption of seafood and aquatic products contaminated with pathogen as well as bacterial toxins (Food and Drug Administration [FDA], 2018). *V. parahaemolyticus* strains carry the *tox R* gene that encodes an important membrane-localized regulatory protein, and it commonly gets involved in the bacterial regulation of a variety of expression products (Lin et al., 1993; Zhang et al., 2018). For example, *tox R* expression is able to regulate the production of thermostable direct hemolysin (TDH), TDH related hemolysin (TRH), T3SS1, and T3SS2 (Whitaker et al., 2012; Hubbard et al., 2016; Mala et al., 2016). TDH with hemolytic activity, enterotoxicity and cytotoxicity, is defined as a type of membrane pore protein. TRH, a heat labile toxin, is thought to have a similar hemolytic activity and pathogenic mechanism to TDH (Matsuda et al., 2010; Leoni et al., 2016). However, recent researches even showed that some clinical *V. parahaemolyticus* strains do not carry both major virulence factors but remains pathogenic indicating putative virulence factors exist, and pathogenicity might be achieved with different strategies employed by different strains (Mahoney et al., 2010; Jones et al., 2012; Cai and Zhang, 2018). To date, *tox R*, *tdh*, and *trh* gene sequences have been identified in *V. parahaemolyticus* isolates using PCR-based methods (Shirai et al., 1990; Xie et al., 2017).

The molecular identification and classification of *Vibrio parahaemolyticus* usually used PCR technology and Sanger sequencing to analyze the core genes. For example, based on the variations in somatic O and capsular K antigens, *V. parahaemolyticus* can be classified into 13 O and 71 K serogroups (Iguchi et al., 1995). It’s believed that various serogroups, associated with high virulence, have been identified in environmental isolates and clinical samples (Mala et al., 2016; Li et al., 2018; Guin et al., 2019). Additionally, the multilocus sequence typing (MLST) scheme, was originally proposed for the identification of closely related bacterial genotypes. However, the genealogical information derived from the DNA sequences also allowed one to address questions about species boundaries and evolutionary relationships (Harismendy et al., 2009; Perez-Losada et al., 2013; Chen et al., 2015; Chen and Perfect, 2017). Both O-serogroup typing and MLST scheme is easy to operate, quick, and facilitates the exchange of data between laboratories via public databases, so that it can be used to investigate the source of infection and route of transmission.

*Vibrio parahaemolyticus* strains were frequently detected and isolated from seafood and aquatic products. Recently, this bacterium with antibiotic resistant phenotype has also been present in RTE food (Cho et al., 2016; Mala et al., 2016). As a result of an increase in the quality of life in China, RTE food has become a product of mass consumption. This study mainly aims to investigate the seasonal prevalence of *V. parahaemolyticus* from fish, shrimp, and RTE food in China. We also characterized the prevalence of each isolate and combined phenotyping (antibiotic resistance patterns) and genotyping (MLST) methods to determine and analyze the genetic relatedness and diversity among the tested bacterial isolates. These findings may facilitate the evaluation of the microbiological profiles of edible products and contribute to the effort to ensure food safety in China.

## Materials and Methods

### Sample Collection

**FIGURE 1:**
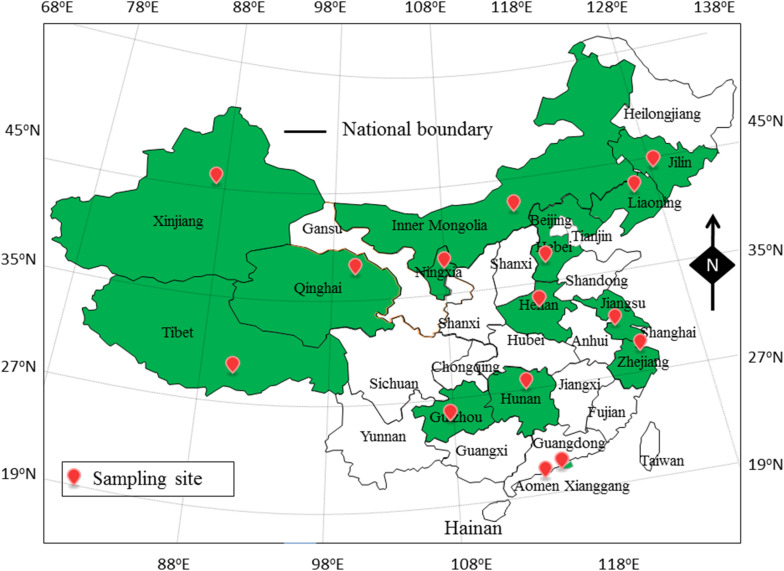
The sampling site of foods sample in China.

In this study, 905 food samples, including 300 RTE food samples, 240 fish samples and 365 shrimp samples, were collected from retail stores in 15 cities of 15 provinces of China (Figure 1). The climate of sample collection was cold from September 2015 to March 2016 (winter), and hot from March 2016 to September 2016 (summer). All of food samples were collected and placed in sterile sealed plastic bags from environmental microbial contamination, and stored in a cold box at <4°C during transportation. Sample processing and quantification of *V. parahaemolyticus* were performed immediately. Two *V. parahaemolyticus* reference strains, ATCC 33847 (O4, *tox R^+^*, *tdh*^+^, *trh*^–^) and ATCC 17802 (O1, *tox R^+^*, *tdh*^–^, *trh*^+^), were obtained from the American Type Culture Collection (ATCC; Manassas, VA, United States).

### Sample Processing, Quantification, and Isolation of *V. parahaemolyticus*

Sample processing, bacterial load, and qualitative detection of *V. parahaemolyticus* in the samples were performed, according to the National Food Safety Standards of China Document GB4789.7-2013 with minor modifications. In brief, 25 g of each sample were placed into 225 mL of alkaline peptone water (APW) containing 3% NaCl (Huankai, Guangzhou, China). It was suggested that fish and shrimp samples were taken from surface tissues, intestines or gills. Then, 1 mL suspension was collected from the top 1 cm of each tube, a serial dilution was prepared up to 10^3^, and 1 mL of each dilution was transferred into a new tube with 9 mL of APW (3% NaCl)., The enumeration of presumptive *V. parahaemolyticus* was determined by the most probable number (MPN) method of culture tubes positive for *V. parahaemolyticus* to MPN/g using an MPN table.

Purification and identification of suspected *V. parahaemolyticus* was performed after 18 h of incubation at 37°C in APW (3% NaCl). To form *V. parahaemolyticus* colonies appeared green or blue green, a loopful from culture medium was streaked on thiosulfate-citrate-bile salts-sucrose (TCBS) agar plates (Huankai, Guangzhou, China) and all plates were incubated at 37°C under aerobic conditions for 18–24 h. One to three forming colonies were purified by streaking onto Chromogenic *Vibrio* Medium (Huankai, Guangzhou, China) and incubated at 37°C under aerobic conditions for 24 h. Each mauve colony from each Chromogenic *Vibrio* Medium plate was picked for identification tests including halophilism tests, oxidase activity assessment, Gram staining, the 3.5% NaCl triple-sugar-iron (TSI) test, and API 20E diagnostic strips testing (BioMerieux Company, Marcyl’Étoile, France).

### Antimicrobial Susceptibility Testing

A total of 202 *V. parahaemolyticus* isolates were subjected for antimicrobial susceptibility testing by disk-diffusion method, according to the detailed guidelines of the Clinical and Laboratory Standards Institute (CLSI, 2018). Nutrient Agar (Huankai, Guangzhou, China), Muller-Hinton agar (Huankai, Guangzhou, China) and a panel of 12 antibiotic disks (Oxoid, Hampshire, United Kingdom) were used for antibiotic susceptibility tests. *V. parahaemolyticus* isolates were inoculated on Nutrient Agar and cultivated overnight at 37°C. Each bacterial culture was adjusted to the turbidity of a 0.5 McFarland standard and spread onto Muller-Hinton agar plates. After that, the antibiotic disks were placed on the plates that were then incubated for 18 h at 37°C. The following 12 antimicrobial disks were used in this study (with concentrations per disk given in parentheses): ampicillin (10 μg), azithromycin (15 μg), cefazolin (30 μg), cephalothin (30 μg), chloramphenicol (30 μg), ciprofloxacin (5 μg), gentamicin (10 μg), kanamycin (30 μg), nalidixic acid (30 μg), streptomycin (10 μg), trimethoprim-sulfamethoxazole (25 μg), or tetracycline (30 μg). The results of diameter of the inhibition zone around each disk were recorded and expressed as sensitive (S), intermediate (I), and resistant (R), following the methods of the CLSI. *Escherichia coli* ATCC 25922 and *V. parahaemolyticus* ATCC 17802 were used as quality control organisms.

### Detection of Virulence Genes: *tox R*, *tdh*, and *trh*

Bacterial whole genome was extracted from cell pellet using a commercial Universal DNA Extraction Kit (Sangon, Shanghai, China), according to the manufacturer’s formal instructions. Following DNA extraction, the detection of three virulence genes (*tox R*, *tdh*, and *trh*) in all 202 *V. parahaemolyticus* isolates was determined through PCR. The primer sequences of *tox R* were *tox R*-F: GTCTTCTGACGCAATCGTTG, *tox R*-R: ATACGAGTGGTTGCTGTCATG (Sangon, Shanghai, China) (Cho et al., 2016; Mala et al., 2016). Detection of the *tdh* and *trh* genes was performed as described previously (West et al., 2013), using the primers *tdh*-F: CTGTCCCTTTTCCTGCCCCCG, *tdh*-R: AGCCAGACACCGCTGCCATTG; *trh*-F: ACCTTTTCCTT CTCCWGGKTCSG, and *trh*-R: CCGCTCTCATATGCYTCG ACAKT (Sangon, Shanghai, China). PCR reagents (total volume, 25 μL) were prepared by mixing 12.5 μL of 2 × PCR Mix (Qiagen), 0.5 μL forward primer, 0.5 μL reverse primer, 0.5 μL genomic DNA, and 11 μL ddH_2_O. All genes were amplified in a Bio-Rad PTC-200 Thermal Cycler (Bio-Rad, Hercules, CA, United States) using the following PCR protocol: pre-denaturation at 95 °C for 5 min; 40 cycles of 94 °C for 1 min (denaturation), 62 °C for 1 min (annealing), 72 °C for 1 min (extension), and a final extension of 72 °C for 2 min. Before Images were performed in a Gel Image system (Bio-Rad, Hercules, CA, United States), all amplified products were visualized by 2% agarose gel containing GoldView. To validate the PCR performance, whole genome from *V. parahaemolyticus* strains ATCC33847 (*tdh*^+^) and ATCC17802 (*trh*^+^) were used as positive control templates, and sterile purified water was used as the negative control.

### Multiplex PCR Test for O Serogroups

The serogroups of *V. parahaemolyticus* isolates were identified using the multiplex PCR technique. The specific primer sequences and PCR protocol were set as described previously (Chen et al., 2012).

### MLST Analysis

Multilocus sequence typing analysis was performed using seven conserved housekeeping genes (i.e., *dnaE*, *gyrB*, *recA*, *dtdS*, *pntA*, *pyrC*, and *tnaA*) to characterize diversity and epidemiology (Xu et al., 2017). We randomly chosed 108 isolates of *V. parahaemolyticus* from 202 food-borne isolates. PCR reagents (total volume, 50 μL) were prepared by mixing 5.0 μL of 10× PCR buffer (Takara, Dalian, China), 1 μL forward primer, 1 μL reverse primer, 1 μL genomic DNA, and 42 μL ddH_2_O. The PCR amplification conditions in a Bio-Rad PTC-200 Thermal Cycler were as follows: a cycle of pre-denaturation at 94 °C for 5 min; 35 cycles at 94 °C for 30 s (denaturation), 55 °C for 30 s (annealing), 72 °C for 2 min (extension), and a final extension at 72 °C for 10 min. Before sequenced on an ABI 3730 sequencer (Applied Biosystems), the target amplification products were purified using a PCR purification kit (Qiagen, Germany). To obtain allele numbers and define sequence types (STs), the sequences were analyzed on the MLST database^1^. Genotyping analysis and phylogenetic analysis were based on MLST sequences. Both phylogenetic tree and minimum spanning tree were generated by BioNumerics 7.6.

## Results

### Prevalence and Bacterial Load Determination of *V. parahaemolyticus*

Of the 905 food samples, 101 (11.16%) were positive for *V. parahaemolyticus*. This included 34 (14.17%) of the 240 fish samples, 56 (15.34%) of the 365 shrimp samples, and 11 (3.67%) of the 300 RTE food samples. Overall, the density of *V. parahaemolyticus* varied from 1.50 to 100 MPN/g. Of the positive samples, 64.36% contained a bacterial load of 3 to 10 MPN/g, 18.81% contained a load >3 MPN/g, and 16.83% samples exceeded 10 MPN/g (Table 1).

**TABLE 1:** Prevalence and levels of *Vibrio parahaemolyticus* in Chinese samples.

Type of samples	No. of samples analyzed	No. of samples positive (%)	No. of samples containing the pathogen (MPN/g)		
			
			<3	3 to 10	>10 to 10^2^
Fish	240	34 (14.17)	6	22	6
Shrimp	365	56 (15.34)	11	35	10
RTE Foods	300	11 (3.67)	2	8	1
Total	905	101 (11.16)	19	65	17

Analysis to determine the seasonal prevalence of the pathogen revealed a higher number of positive samples in the summer compared to winter (Table 2). The rate of *V. parahaemolyticus* detection in summer reached 14.87%, while it only reached 7.26% in winter. Besides, the prevalence of *V. parahaemolyticus* in summer were 20.83% (fish samples), 20.11% (shrimp samples), and 4.38% (RTE food samples). On the other hand, the prevalence in winter was 6.67% (fish samples), 12.15% (shrimp samples), and 1.40% (RTE food samples). It meant the contamination level of all samples in summer was higher than that observed in the winter samples Moreover, the mean level of *V. parahaemolyticus* in samples collected during summer and winter were 8.08 and 5.3 MPN/g, respectively. Therefore, the population of *V. parahaemolyticus* was certainly abnormal for samples collected in different seasons. In total, 202 potential *V. parahaemolyticus* isolates from 101 samples were detected and purified for further bacterial classification and epidemiology.

**TABLE 2:** Prevalence and levels of *Vibrio parahaemolyticus* in Chinese samples during different seasons.

Samples	No. of samples analyzed	No. of samples positive (%)	No. of samples containing the pathogen (MPN/g)		
			
			<3	3 to 10	>10 to 10^2^
Summer	464	69 (14.87)	8	49	12
Winter	441	32 (7.26)	11	16	5
Total	905	101 (11.16)	19	65	17

### Antimicrobial Susceptibility of the *V. parahaemolyticus* Isolates

The extent of antibiotic resistance was examined in 202 isolates of *V. parahaemolyticus*. And bacteria exhibited three or more resistant phenotypes was called multidrug-resistant *V. parahaemolyticus*. The antimicrobial resistance profiles of the isolates are shown in Table 3 and antibiotic-resistant phenotypes of all isolates are summarized in Supplementary Table S1. The isolates were mostly resistant to ampicillin, with 79.20% R ratings and 14.36% I ratings. Additionally, the isolates exhibited relatively high resistance rates of 74.75%, 65.84%, 58.91%, and 44.55%, to cephalothin, streptomycin, cefazolin, and kanamycin, respectively. However, most of the examined isolates were susceptible to nalidixic acid (97.52%), ciprofloxacin (96.04%), and chloramphenicol (90.59%). Among the remaining tested antibiotics, the next highest susceptibility rates were observed for tetracycline (78.71%), trimethoprim-sulfamethoxazole (78.22%), and azithromycin (77.23%). In addition, among all isolates, three were multidrug-resistant (843, 860, and 2928A2), showing resistance to nine antibiotics, and seven isolates showing resistance to eight antibiotics. Of the isolates, 73.27% were resistance to more than three antibiotics.

**TABLE 3:** Antimicrobial resistance profiles of *Vibrio parahaemolyticus* isolates from China.

Antimicrobial agents	*Vibrio parahaemolyticus*(*n* = 150)			Zone diameters (mm)		
		
	No. (%) of R	No. (%) of I	No. (%) of S	*R*	*I*	*S*
Ampicillin (AMP)	160 (79.20)	29 (14.36)	13 (6.44)	≤13	14–16	≥17
Azitromycin (AZM)	26 (12.87)	20 (9.90)	156 (77.23)	≤13	14–17	≥18
Cefazolin (KZ)	119 (58.91)	63 (31.19)	20 (9.90)	≤14	15–17	≥18
Cephalothin (KF)	151 (74.75)	33 (16.34)	18 (8.91)	≤14	15–17	≥18
Chloramphenicol (C)	8 (3.96)	11 (5.45)	183 (90.59)	≤12	13–17	≥18
Ciprofloxacin (CIP)	7 (3.46)	1 (0.50)	194 (96.04)	≤15	16–20	≥21
Gentamicin (CN)	42 (20.79)	48 (23.76)	112 (55.45)	≤12	13–14	≥15
Kanamycin (K)	90 (44.55)	103 (50.99)	9 (4.46)	≤13	14–17	≥18
Nalidixic acid (NA)	5 (2.48)	0 (0.00)	197 (97.52)	≤13	14–18	≥19
Streptomycin (S)	133 (65.84)	54 (26.73)	15 (7.43)	≤11	12–14	≥15
Trimethoprim-sulfamethoxazole (SXT)	21 (10.40)	23 (11.39)	158 (78.22)	≤10	11–15	≥16
Tetracycline (TE)	29 (14.36)	14 (6.93)	159 (78.71)	≤14	15–18	≥19

**TABLE 4:** Distribution of the tox R tdh and trh genes in V. parahaemolyticus isolates.

Sample category	No. of isolates	*tox R*-positive (%)	*tdh*-positive (%)	*trh*-positive (%)
Fish	64	100 (64/64)	15.63 (10/64)	18.75 (12/64)
Shrimp	123	100 (123/123)	7.32 (9/123)	21.95 (27/64)
RTE Foods	15	100 (15/15)	6.67 (1/15)	6.67 (1/15)
Total	202	100 (202/202)	9.90 (20/202)	19.80 (40/202)

### Detection of *tox R*, *tdh*, and *trh* Genes in *V. parahaemolyticus* Isolates

All of the 202 *V. parahaemolyticus* isolates were tested for the presence of *tox R*, *trh* and *tdh*, and the results are shown in Supplementary Table S1. All of the isolates were positive for the *tox R* gene. Among these, 9.90 and 19.80% of the *V. parahaemolyticus* strains carried the *tdh* or the *trh* genes, respectively, whereas eight isolates harbored both the *tdh* and *trh* genes. Among eight isolates (*tdh*^+^, *trh*^+^), 566, 654, 690, 3154B2, and 709B1 were form shrimp samples whereas the other three were (3331A1, 3478B2, and 3481B3) from fish samples. Lastly, the rates of *tdh*-positive strains among fish, shrimp, and RTE foods were 15.63, 7.32, and 6.67%, respectively. The rates of *trh* gene positivity were 18.75, 21.95, and 6.67%, respectively.

### O-Serogroup Analysis

The analysis of multiplex PCR-ebased O serogroups is performed to obtain information of DNA fragment size on the distribution of bacterial classification among the 202 *V. parahaemolyticus* isolates. The results are shown in Table 5. With the exception of serogroups O3, O8, and O9, all other serogroups were detected among the isolates. O2 serogroup was the most prevalent (49.01%), followed by O1 serogroup (22.77%). Besides, the occurrence of *V. parahaemolytiucs* (O2) were 50.00% (32 of 64 strains from fish samples), 51.22% (63 of 123 strains from fish samples) and 26.67% (4 of 15 strains from RTE food samples). Moreover, there were seven cities (Guiyang, Hangzhou, Hohhot, Hongkong, Macao, Urumchi, and Zhengzhou) whose main prevalent of serogroup was O2 and the occurrence was 52.27, 42.85, 57.89, 67.58, 50.00, 62.50, and 100.00%. The serogroups of *V. parahaemolyticus* ATCC17802 and ATCC33847, were O1 and O4, respectively.

**TABLE 5:** Results of the PCR-based O-antigen serotyping of *Vibrio parahaemolyticus* isolates.

Serogroup		Product size (bp)	No. (%) of isolates analyzed
Group 1	O1	474	46 (22.77)
	O2	238	99 (49.01)
	O4	671	1 (0.50)
	O5	852	1 (0.50)
	O6	1409	4 (1.98)
	O10	343	13 (6.44)
Group 2	O3	868	0 (0.00)
	O7	385	1 (0.50)
	O8	680	0 (0.00)
	O9	419	0 (0.00)
	O11	524	5 (2.48)
	O12	256	21 (10.40)
Uncertain			11 (5.45)
Total			202

### MLST

**FIGURE 2:**
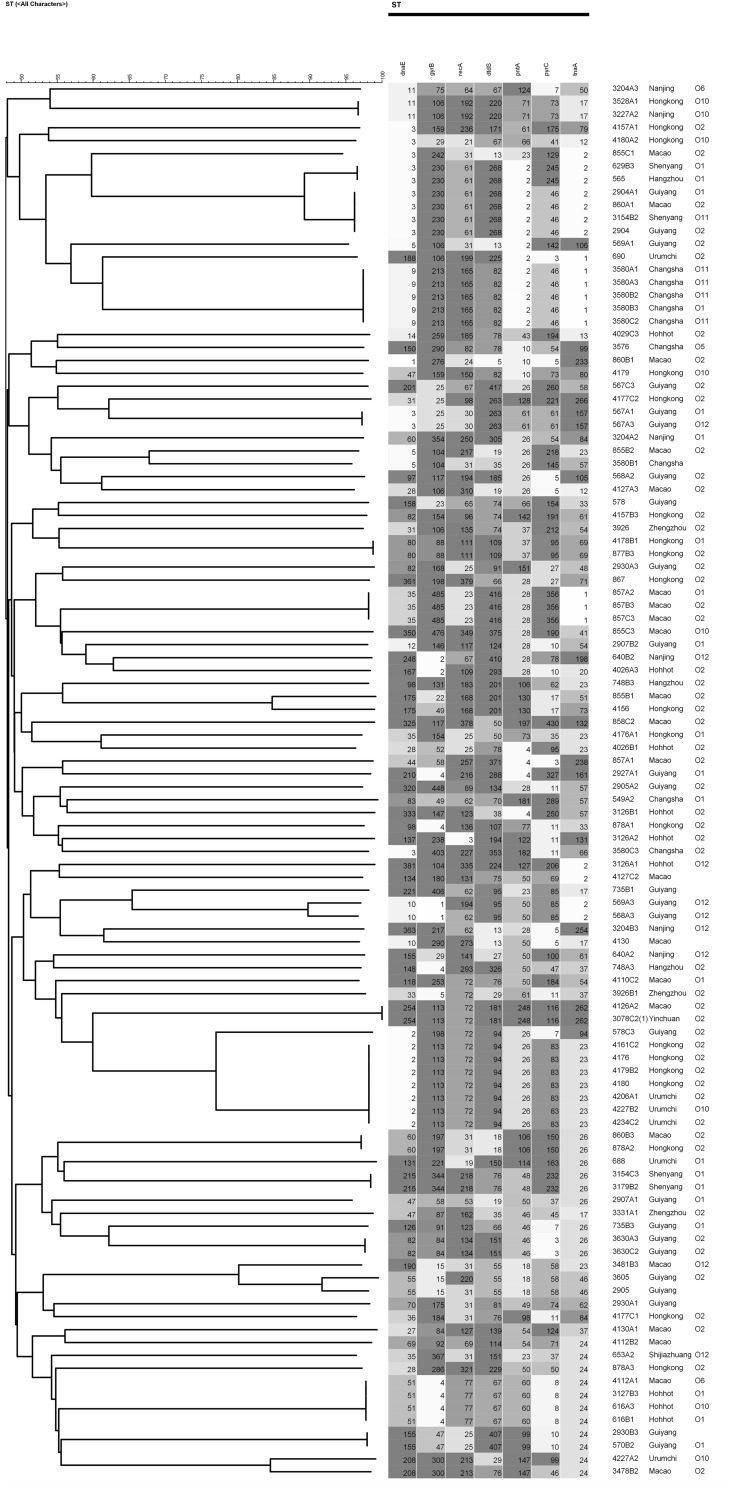
MLST results about serogroup of 108 isolates of *V. parahaemolyticus.*

**FIGURE 3:**
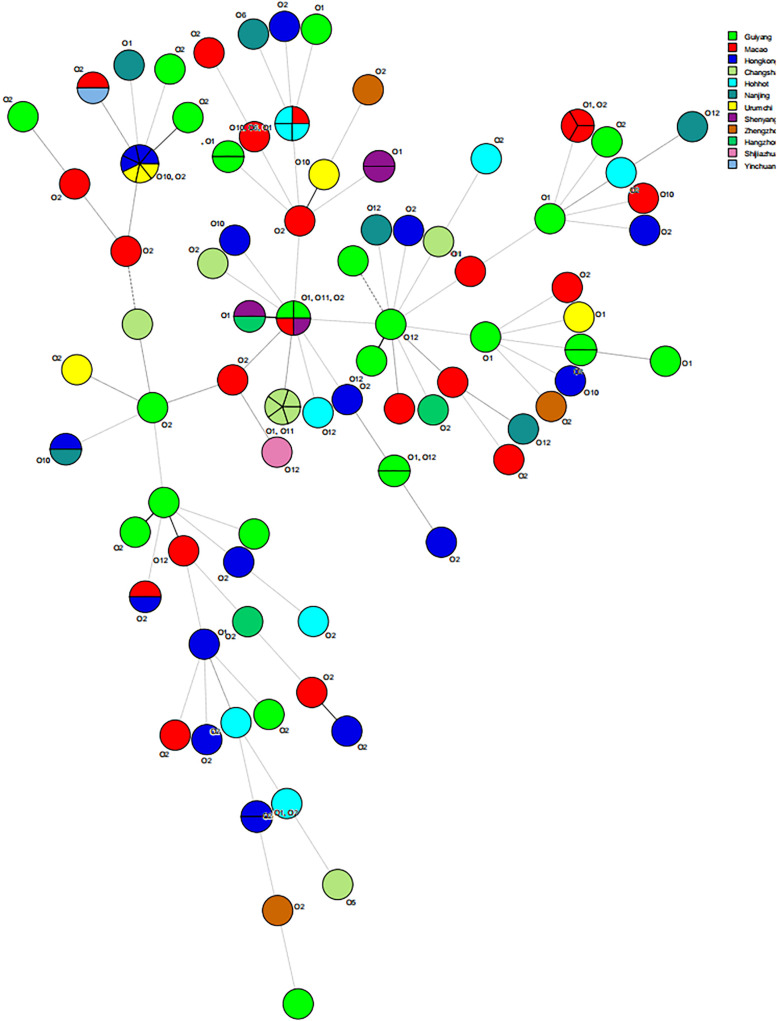
Minimum spanning tree results about source of 108 isolates of *V. parahaemolyticus.*

The results of MLST of the 108 *V. parahaemolyticus* isolates tested are shown in Table 6, Figures 2, 3. Our research revealed that 81 different STs were identified in 108 isolates derived from fish, shrimp and RTE-food samples (Table 6). In addition, 40 of them were searched in pubMLST Database. Moreover, ST411 (6.5%), ST992 (4.6%), ST423 (3.7%), ST693 (3.7%), and ST1352 (2.8%) were the most common STs detected in this study. More importantly, 41 new STs, including 46 strains, were identified in tested isolates. Lastly, the minimum spanning tree based on allele numbers and O-serogroup typing of the isolates did not reveal a clear clustering pattern connected with the serogroup emergence.

**TABLE 6:** MLST typing results of 108 isolates of *V. parahaemolyticus*.

ST	Number(%)	ST	Number(%)
114	1 (0.93)	864	2 (1.85)
162	1 (0.93)	846	1 (0.93)
212	1 (0.93)	891	1 (0.93)
217	2 (1.85)	965	1 (0.93)
243	1 (0.93)	992	5 (4.63)
253	1 (0.93)	1010	1 (0.93)
283	1 (0.93)	1070	1 (0.93)
342	1 (0.93)	1087	1 (0.93)
373	1 (0.93)	1249	1 (0.93)
411	7 (6.48)	1256	2 (1.85)
423	4 (3.70)	1352	2 (1.85)
445	1 (0.93)	1353	1 (0.93)
452	1 (0.93)	1592	1 (0.93)
456	1 (0.93)	1694	1 (0.93)
490	1 (0.93)	1825	1 (0.93)
511	1 (0.93)	1829	2 (1.85)
525	2 (1.85)	1,843	1 (0.93)
543	1 (0.93)	1,949	1 (0.93)
564	1 (0.93)	2,061	1 (0.93)
693	4 (3.70)	New	46 (42.59)
863	1 (0.93)		

## Discussion

In our study, samples collection, processing and quantification were based on China Document GB4789.7-2013. Meanwhile, *V. parahaemolyticus* isolates were purified by TCBS agar plate and Chromogenic *Vibrio* Medium. Our results revealed that these methods were fit to isolation and purification of *V. parahaemolyticus* specifically. According to epidemiological data, fish and shrimp may suffer from a severe microbial contamination associated with *V. parahaemolyticus*. Here, we analyzed 300 RTE food, 240 fish and 365 shrimp samples collected in China. From 101 positive samples collected in summer and winter, 202 *V. parahaemolyticus* isolates were detected, purified and identified. As is showed in Table 1, the most severely contaminated edible was shrimp (15.34%). However, the contamination detection rate was lower than that conveyed in other studies that report a prevalence of 28.0% (Caburlotto et al., 2016). The reason may be that the cities were sample collection was conducted were far away from the ocean, so these types of aquatic products were more scarce. Even though the contamination rate was low, a report indicated that the prevalence of *V. parahaemolyticus* in aquatic products is an important cause of food poisoning in Shanghai, China (Zhang et al., 2017). On the other hand, the *V. parahaemolyticus* contamination rate has remained stable at a relatively high level in Shandong and Wenzhou in China (Zhang et al., 2016; Guo et al., 2018). Therefore, to prevent food-borne diseases, it is necessary to highlight the need for microbiological risk assessments of food safety and enhance active monitoring efforts, especially the sanitary management of seafood, aquatic products and RTE food.

Notably, the prevalence of *V. parahaemolyticus* in summer (14.87%) was higher than that in winter (7.26%), and the mean level as well as population of *V. parahaemolyticus* was certainly different for samples collected during the winter and summer. The contamination level in the summer was higher than in the winter. This distinction may be related to the differences in the average temperature change between the seasons, as it was in agreement with the results reported in previous studies that showed a seasonal variation in the occurrence of this pathogen (Parveen et al., 2008; Caburlotto et al., 2016; Zhang et al., 2017). Thus, considering temperature as a factor may enhance the efforts of food quality control, in terms of *V. parahaemolyticus* decontamination, as it is a confirmed pathogen in the WHO risk assessment^2^. As these results were obtained from a large number of variable samples and from most regions in China, the data presented is more representative of China as a whole.

The continuous and extensive abuse of antibiotics in humans as well as animals has led to the urgent state of the outbreak of multidrug-resistant *V. parahaemolyticus* strains worldwide. In our study, high resistance rate of 202 *V. parahaemolyticus* isolates was observed for some antibiotics, such as ampicillin (79.20%), cephalothin (74.75%), and streptomycin (65.84%). Similarly, the occurrence and outbreak of streptomycin- and ampicillin-resistant *V. parahaemolyticus* isolates were also reported in the past 5 years (Hu and Chen, 2016; Jiang et al., 2019). Besides, antibiotic susceptibility tests displayed the highest level of resistance to ampicillin, signifying that ampicillin may be ineffective and invalid for the treatment of *Vibrio* sp. infections (Hu and Chen, 2016; Jiang et al., 2019). More importantly, ampicillin resistance has been reported to be 100% in other studies (Fattel et al., 2019). Such ampicillin-resistant pattern was closely related to human behavior, such as the application of first generation antibiotics including ampicillin in aquaculture of *Vibrio* infection. More seriously, some isolates even revealed antibiotic resistant phenotype to gentamicin, tetracycline, or ciprofloxacin, which are first-line antibiotics widely used in clinical treatment for bacterial infection (Elmahdi et al., 2016; Tan et al., 2017). At the same time, 73.27% of 202 strains with multi-drug resistant phenotype were detected and three of them showed resistance to nine antibiotics. The rate was higher than in previous reports (Letchumanan et al., 2015a; Xu et al., 2016). Such observation may be closely related to the abuse of various antibiotics to prevent and control pathogenic bacterial infections in aquatic environments (Cabello et al., 2013). Generally, it is important to evaluate mutation and evolution of resistance determinants in *V. parahaemolyticus*, as infection caused by emerging of multi-drug antimicrobial-resistant strains plays an essential role in clinical treatment. Recently, the Food and Agriculture Organization (FAO) has designed action plans to increase awareness regarding the urgent state of drug-resistant food-borne pathogens and promote prudent use of antimicrobials^3^. Moreover, research focusing on providing alternatives to antibiotics is urgently needed, not only for disease control, but also for the sustainable development of the aquaculture industry.

PCR assay for detection of bacterial virulence genes is useful, rapid and efficient. The *tox R* gene, which mainly gets involved in the regulation of many other genes, such as bacterial persistence, biofilm formation and virulence, has been detected ubiquitously in *V. parahaemolyticus*. Letchumanan et al. (2015b) discovered that 57.8% (185/320) *V. parahaemolyticus* isolates carried *tox R* gene. Kang et al. (2017) detected 31 of the 44 isolates were positive *V. parahaemolyticus* strains for *tox R* gene. Thus, it is necessary and urgent to obtain a deeper understanding of the regulatory mechanism of *tox R* serving as activator of lethality and enterotoxicity. It is well-known that *V. parahaemolyticus*, especially clinical strains, expressed TDH and TRH encoded by hemolysin genes (*tdh* and *trh*) are believed to induce inflammatory gastroenteritis rapidly (Mahoney et al., 2010; Ceccarelli et al., 2013; Raghunath, 2015; Chen et al., 2018). Thus, detecting the hemolysin genes through PCR assay could be primary but efficient method to infer the virulence potential of food derived isolates. In our study, 9.90 and 19.80% of the strains were positive for *tdh* and *trh* gene indicating the expression of TDH and TRH exist. Our findings were more serious than those reported previously on *V. parahaemolyticus* isolates from fish, shrimp and RTE food (Xie et al., 2016). The evolution of *tdh* or *trh* isolates may be affected by environmental factors, including interaction with other hosts (Wilson and Salyers, 2003). The evolution of *tdh* or *trh* isolates, affected by environmental factors and interacted with other hosts, represents a possible risk to public health. Therefore, monitoring of *V. parahaemolyticus* pathogenic factors are important to protect aquatic products and RTE food in sales chain and improve food safety in the industry.

O antigen, one of three distinct regions, is an important component of lipopolysaccharide, such as *Escherichia coli* and *Salmonella enterica* (Samuel et al., 2004). Depending on external factors, *V. parahaemolyticus* can produce a capsule with the variability of the O antigen and it was believed as a primary information of bacterial isolates classification. Our data indicated that the serovar O2 (49.01%) was the predominant serogroup in fish (50.00%), shrimp (51.22%), and RTE food (26.67%). Such finding was not in accordance with a previous study that identified the O3 serogroup as the predominant serogroup contaminating shellfish in the eastern coast of China (Zhao et al., 2011). The next highest prevalent was O1 serogroup (22.77%), which may become an epidemic strain. Monitoring serogroup variation could be an effective in improving our understanding of *V. parahaemolyticus* isolates that cause food poisoning. Besides, The O antigen of *Vibrio* sp., as a receptor, located on the bacterial surface and made host a major target of specific phages (Seed et al., 2012; Xu et al., 2013). The prevention and control of multi-drug resistant bacteria by phages targeting *Vibrio parahaemolyticus* O serogroup may be a novel and efficient strategy.

Recently, molecular subtyping has been increasingly used for the analysis of genetic diversity. The MLST method, commonly considered to be molecular typing, can be classified in two basic strategies. In this study, one of these was employed, that relied on allele and ST determination to estimate relatedness among 108 *V. parahaemolyticus* isolates. On one hand, MLST data can contribute to species discrimination, as it provides both genealogical information and information on recombination, which is critical to *V. parahaemolyticus* identification. The results showed 81 STs, of which 41 (50.62%) STs, composed of 46 strains, were newly identified and revealed a high degree of diversity among the strains tested. A number of reports have shown similar results. Urmersbach et al. (2014) reported that 130 *V. parahaemolyticus* strains obtained from a marine environment were classified into 82 STs, and 82.9% of them had not been recorded in the pubMLST database previously. Besides, Lopatek et al. (2018) identified 51 novel STs in the isolates from different species of seafood available on the Polish market that originating from various countries. Moreover, a total of 68 STs were identified in the 90 *V. parahaemolyticus* isolates, and 41 (60.3%) of them were considered novel (Jiang et al., 2019). Both our study and the research conducted previously have revealed how poorly the current pubMLST data set represents the diversity within *V. parahaemolyticus*. Therefore, efficient strain identification and accurate information presented in the public database are essential to understand the processes of transmission, perform epidemiological surveillance and subsequently the design of public health control strategies (Comas et al., 2009).

## Conclusion

Diarrhea caused by the food-borne pathogen, *V. parahaemolyticus*, has been a long-standing problem. In summary, this is a comprehensive study that describes the prevalence, serogroup, virulence genes, antibiotic resistant phenotype, molecular classification, and genetic diversity of *V. parahaemolyticus* detected in aquatic products and RTE foods in China in summer and winter. Significantly, the prevalence of *V. parahaemolyticus* was inconsistent between summer (14.87%) and winter (7.26%). This study shows the percentage of the isolates that possess the *tdh* and *trh* genes, being 9.90 and 19.80%, while there were eight of 202 isolates carried both genes, respectively. More seriously, all isolates were tested positive for *tox R* gene. In addition, O2 serogroup was found to be the most prevalent of *V. parahaemolyticus* isolated mainly from seven cities. Moreover, *V. parahaemolyticus* isolates exhibited resistance to ampicillin, cephalothin and streptomycin were widespread. Lastly, both MLST phylogenetic analysis and MST, basing on 41 novel STs and 40 reported STs, showed large genetic diversity of *V. parahaemolyticus* tested. As aquatic products and RTE foods consist of popular food choices in China, our findings may be useful in guiding appropriate monitoring strategy, improving the understanding of antimicrobial susceptibility patterns, and providing information for the assessment of exposure to *V. parahaemolyticus* during food consumption, which are vital to ensure the safety of such products and safeguard human health.

## Data Availability Statement

All datasets generated for this study are included in the article/Supplementary Material.

## Author Contributions

QW, YL, TX, RP, and JZ conceived and designed the experiments. YL, YZ, and TX performed the experiments. RP, TL, HZ, HW, and LX analyzed the data. JW, YD, MC, and SW contributed reagents, materials, and analysis tools. YL, TX, QW, and XW contributed to the writing of the manuscript. All authors contributed to the article and approved the submitted version.

## Conflict of Interest

The authors declare that the research was conducted in the absence of any commercial or financial relationships that could be construed as a potential conflict of interest.

## Footnotes

1 https://pubmlst.org/vparahaemolyticus

2 https://www.who.int/publications/i/item/microbiological-risk-assessment-series

3 http://www.fao.org/3/a-i4296t.pdf
