# Supplementary material for: Food-Borne Vibrio parahaemolyticus in China: Prevalence, Antibiotic Susceptibility, and Genetic Characterization
Source: Front Microbiol. 2020 Jul 16;11:1670. doi: 10.3389/fmicb.2020.01670 (PMC7378779; doi:10.3389/fmicb.2020.01670)
Supplement: Supplementary file 1 [file Table_1.docx]

# Results of antimicrobial resistance, serotyping, and virulence genes of *V. parahaemolyticus* isolates in this study

| No. | Strain | Source | Location | Resistance profileb | Serogroup | *tox R* | *tdh* | *trh* |
| --- | --- | --- | --- | --- | --- | --- | --- | --- |
| 1 | 565 | fresh shrimp | Hangzhou | S-KF-AMP-K-CN-KZ | O1 | + | - |  |
| 2 | 566 | fresh shrimp | Hangzhou | S-KF-AMP-CN-KZ | O1 | + | + | + |
| 3 | 567 | fresh shrimp | Guiyang | S-KF-AMP-TE-C-K | O1 | + | - | - |
| 4 | 568 | fresh shrimp | Guiyang | KF-AMP-K-KZ | O2 | + | - | - |
| 5 | 578 | shelled shrimp | Guiyang | KF-AMP | uncertain | + | - | - |
| 6 | 654 | fresh shrimp | Shijiazhuang | S | O12 | + | + | + |
| 7 | 688 | fresh shrimp | Urumchi | AMP-K | O1 | + | - | + |
| 8 | 690 | fresh shrimp | Urumchi | KF-AMP-TE | O2 | + | + | + |
| 9 | 706 | shelled shrimp | Macao | S-KF-AMP-K | O12 | + | - | + |
| 10 | 735 | fresh shrimp | Guiyang | AMP | O1 | + | - | - |
| 11 | 749 | fresh shrimp | Hangzhou | S-KF-AMP-KZ | O2 | + | - | - |
| 12 | 831 | fresh shrimp | Hohhot | S-KF-AMP-TE-C-K-CN-KZ | O2 | + | - | - |
| 13 | 843 | fresh shrimp | Changchun | S-KF-AMP-TE-K-CN-AZM-SXT-KZ | O2 | + | - | - |
| 14 | 855 | fresh shrimp | Macao | S-KF-AMP-TE-K-KZ | O2 | + | - | - |
| 15 | 860 | shelled shrimp | Macao | S-KF-AMP-TE-C-K-CN-AZM-KZ | O2 | + | - | - |
| 16 | 863 | shelled shrimp | Macao | KF-AMP-K-CN-KZ | O2 | + | - | - |
| 17 | 867 | fresh shrimp | Hongkong | S-AMP-KZ | O2 | + | - | - |
| 18 | 2807 | fish | Changsha | S-KF-AMP-K-KZ | O1 | + | - | - |
| 19 | 2904 | fish | Guiyang | S-KF-AMP-KZ | O2 | + | - | - |
| 20 | 2905 | fish | Guiyang | S-KF-K-CN-AZM | uncertain | + | - | - |
| 21 | 2927 | fresh shrimp | Guiyang | KF-AMP-KZ | O2 | + | - | - |
| 22 | 3126 | fish | Hohhot | S-AMP-SXT | O1 | + | - | - |
| 23 | 3404 | fresh shrimp | Urumchi | KF-AMP-K | O2 | + | - | + |
| 24 | 3457 | fresh shrimp | Macao | AMP-KZ | O12 | + | - | + |
| 25 | 3528 | fish | Hongkong | KF-AMP-KZ | O2 | + | + | - |
| 26 | 3530 | fish | Hongkong | KF-AMP-K | O7 | + | - | - |
| 27 | 3576 | fish | Changsha | S-KF-AMP-K-KZ | O5 | + | - | - |
| 28 | 3605 | fish | Guiyang | KF-AMP | O2 | + | - | - |
| 29 | 3612 | Deli meat | Guiyang | S-KF-AMP-KZ | O2 | + | - | - |
| 30 | 3630 | fresh shrimp | Guiyang | S-KF-AMP-K-KZ | O2 | + | - | - |
| 31 | 3926 | fresh shrimp | Zhengzhou | S-KF-AMP-K-CN-KZ | O2 | + | - | - |
| 32 | 4026 | fresh shrimp | Hohhot | S-KF-AMP-K-CN-AZM-KZ | O2 | + | - | - |
| 33 | 4027 | fish | Hohhot | S-KF-AZM-KZ | O2 | + | - | - |
| 34 | 4127 | fresh shrimp | Macao | S-KFK-AMP-KZ | O6 | + | - | - |
| 35 | 4130 | fish | Macao | S-KF-AMP-K-CN | uncertain | + | - | - |
| 36 | 4156 | fish | Hongkong | S-KF-CIP-KZ | O2 | + | - | + |
| 37 | 4176 | fish | Hongkong | S-KF-K-KZ | O2 | + | - | - |
| 38 | 4177 | fresh shrimp | Hongkong | S-KF-AMP-TE-K-KZ | O2 | + | - | - |
| 39 | 4179 | fish | Hongkong | S-KF-K-KZ | O10 | + | - | - |
| 40 | 4180 | fish | Hongkong | S-CN | O2 | + | - | - |
| 41 | 2876C2 | fresh shrimp | Hangzhou | S-KF-AMP-K-KZ | O1 | + | - | - |
| 42 | 2904A1 | fish | Guiyang | S-KF-AMP-K-CN-KZ | O1 | + | - | - |
| 43 | 2905A2 | fish | Guiyang | S-KF-AMP-K-CN-AZM-SXT-KZ | O2 | + | - | - |
| 44 | 2907A1 | fish | Guiyang | S-KF-AMP-KZ | O1 | + | - | - |
| 45 | 2907B2 | fish | Guiyang | S-KF-AMP-TE-KZ | O1 | + | - | - |
| 46 | 2926A3 | fresh shrimp | Guiyang | KF-AMP-TE-SXT-KZ | O2 | + | - | + |
| 47 | 2926B1 | fresh shrimp | Guiyang | KF-KZ | O2 | + | - | + |
| 48 | 2926B3 | fresh shrimp | Guiyang | KF-AMP-KZ | O2 | + | - | - |
| 49 | 2926C1 | fresh shrimp | Guiyang | KF-AMP-TE-KZ | O2 | + | - | + |
| 50 | 2927A1 | fresh shrimp | Guiyang | NA-KF-AMP-CIP-TE-SXT-KZ | O1 | + | + | - |
| 51 | 2928A2 | fish | Guiyang | NA-S-KF-AMP-CIP-TE-K-SXT-KZ | O2 | + | + | - |
| 52 | 2930A1 | fish | Guiyang | KF-AMP-TE-AZM-KZ- | uncertain | + | + | - |
| 53 | 2930A3 | fish | Guiyang | S-KF-AMP-KZ | O2 | + | + | - |
| 54 | 2930B3 | fish | Guiyang | S-KF-AMP-CN-AZM-SXT-KZ | uncertain | + | - | - |
| 55 | 3027C1(2) | fresh shrimp | Sining | S-KF-AMP | O2 | + | - | + |
| 56 | 3030A1 | fresh shrimp | Sining | KF-AMP | O12 | + | - | + |
| 57 | 3030A1(2) | fresh shrimp | Sining | KF-AMP-SXT | O12 | + | - | + |
| 58 | 3078C2(1) | fresh shrimp | Yinchuan | S-SXT-KZ | O2 | + | - | - |
| 59 | 3126A1 | fish | Hohhot | S-KF-AMP-K-CN-SXT-KZ | O12 | + | - | - |
| 60 | 3126A2 | fish | Hohhot | S-KF-K | O2 | + | - | - |
| 61 | 3126A3 | fish | Hohhot | 0 | O1 | + | - | - |
| 62 | 3126B1 | fish | Hohhot | S-AMP | O2 | + | - | - |
| 63 | 3126B2 | fish | Hohhot | S-AMP | O2 | + | + | - |
| 64 | 3127B3 | fish | Hohhot | S-KF-AMP | O1 | + | - | + |
| 65 | 3127C3 | fish | Hohhot | S-KF-AMP | O1 | + | - | - |
| 66 | 3128A3 | fresh shrimp | Hohhot | KF-AMP-CIP | O1 | + | - | - |
| 67 | 3154B2 | fresh shrimp | Shenyang | S-AMP-K | O11 | + | + | + |
| 68 | 3154C1 | fresh shrimp | Shenyang | S-KF-AMP-AZM | O1 | + | - | - |
| 69 | 3154C3 | fresh shrimp | Shenyang | S-KF-SXT | O1 | + | - | - |
| 70 | 3179B2 | fish | Shenyang | S-KF-AMP-SXT | O1 | + | - | - |
| 71 | 3204A2 | fresh shrimp | Nanjing | S-K | O1 | + | - | - |
| 72 | 3204A3 | fresh shrimp | Nanjing | S-KF-KZ | O6 | + | - | - |
| 73 | 3204B2 | fresh shrimp | Nanjing | S-KF | O6 | + | - | - |
| 74 | 3204B3 | fresh shrimp | Nanjing | S-AMP | O12 | + | - | - |
| 75 | 3204C1 | fresh shrimp | Nanjing | KF-K | uncertain | + | - | + |
| 76 | 3204C2 | fresh shrimp | Nanjing | AMP-K | O2 | + | - | - |
| 77 | 3226A2 | fish | Nanjing | K | O2 | + | - | - |
| 78 | 3226B2 | fish | Nanjing | AMP | O12 | + | - | - |
| 79 | 3226B3 | fish | Nanjing | KF-AMP-K-KZ | O1 | + | - | - |
| 80 | 3226C1 | fish | Nanjing | 0 | O12 | + | - | + |
| 81 | 3227A2 | fish | Nanjing | KF | O10 | + | - | + |
| 82 | 3331A1 | fish | Zhengzhou | S-K-CN | O2 | + | + | + |
| 83 | 3404B1 | fresh shrimp | Urumchi | KF-SMP | O2 | + | - | + |
| 84 | 3478A2 | fish | Macao | KF-AMP | O1 | + | - |  |
| 85 | 3478B2 | fish | Macao | KF-AMP | O2 | + | + | + |
| 86 | 3478C1 | fish | Macao | S-KF-AMP | O1 | + | + | - |
| 87 | 3478C2 | fish | Macao | KF-AMP-CIP-CN-AZM-KZ | O2 | + | + | - |
| 88 | 3481B3 | fish | Macao | AMP | O12 | + | + | + |
| 89 | 3488B3 | Deli meat | Macao | AMP | O12 | + | - | + |
| 90 | 3505A1 | fish | Hongkong | S-KF-AMP | O2 | + | - | + |
| 91 | 3505B2 | fish | Hongkong | KF | O2 | + | - | + |
| 92 | 3528A1 | fish | Hongkong | S-AMP-K-CN-KZ | O10 | + | - | - |
| 93 | 3528A3 | fish | Hongkong | AMP-KZ | O2 | + | - | - |
| 94 | 3528B3 | fish | Hongkong | AMP-KZ | O12 | + | - | - |
| 95 | 3580A1 | fresh shrimp | Changsha | S-KF-AMP-K-KZ | O11 | + | - | - |
| 96 | 3580A3 | fresh shrimp | Changsha | S-KF-AMP-KZ | O11 | + | - | - |
| 97 | 3580B1 | fresh shrimp | Changsha | S-KF-AMP-K | uncertain | + | - | - |
| 98 | 3580B2 | fresh shrimp | Changsha | S-KF-AMP-CN-AZM-KZ | O11 | + | - | - |
| 99 | 3580B3 | fresh shrimp | Changsha | AMP-CN-KZ | O1 | + | - | - |
| 100 | 3580C2 | fresh shrimp | Changsha | KF-AMP-KZ | O11 | + | - | - |
| 101 | 3580C3 | fresh shrimp | Changsha | AMP-AZM-KZ | O2 | + | - | - |
| 102 | 3630A3 | fresh shrimp | Guiyang | S-KF-AMP-K | O2 | + | - | - |
| 103 | 3630B1 | fresh shrimp | Guiyang | 0 | O2 | + | - | + |
| 104 | 3630B3 | fresh shrimp | Guiyang | S-AMP | O4 | + | - | - |
| 105 | 3630C2 | fresh shrimp | Guiyang | KF-AMP-KZ | O2 | + | - | - |
| 106 | 3926A2 | fresh shrimp | Zhengzhou | S-KF-AMP-TE-K-AZM-SXT-KZ | O2 | + | + | - |
| 107 | 3926B1 | fresh shrimp | Zhengzhou | S-AMP | O2 | + | - | - |
| 108 | 4026A3 | fresh shrimp | Hohhot | S-KF-AMP-KCN-KZ | O2 | + | - | - |
| 109 | 4026B1 | fresh shrimp | Hohhot | S-KF-AMP-TE-C-K-KZ | O2 | + | - | - |
| 110 | 4028A2 | fish | Hohhot | S-KF-AMP-K-KZ | O2 | + | - | - |
| 111 | 4029C3 | fish | Hohhot | AMP-K-CN-KZ | O2 | + | - | + |
| 112 | 4038B3 | Deli meat | Hohhot | KF-AMP-TE-CN-AZM-KZ | O2 | + | - | - |
| 113 | 4110B1 | Deli meat | Macao | AMP-KZ | O1 | + | - | - |
| 114 | 4110C2 | Deli meat | Macao | S-KF-AMP-K-KZ | O1 | + | - | - |
| 115 | 4112A1 | fried rice | Macao | S-KF-AMP-K-KZ | O6 | + | - | - |
| 116 | 4112B2 | fried rice | Macao | S-AMP-K-CN | uncertain | + | - | - |
| 117 | 4112C3 | fried rice | Macao | NA-S-K | O12 | + | - | - |
| 118 | 4126A2 | fish | Macao | S-KF-K-CN-AZM-SXT-KZ | O2 | + | - | - |
| 119 | 4127A3 | fresh shrimp | Macao | S-KF-C-K-KZ | O2 | + | - | - |
| 120 | 4127C2 | fresh shrimp | Macao | S-KF-AMP-C-K-KZ | uncertain | + | - | - |
| 121 | 4128A2 | fish | Macao | KF-AMP-AZM-KZ | O2 | + | - | - |
| 122 | 4130A1 | fish | Macao | S-KF-C-K-CN-AZM | O2 | + | - | - |
| 123 | 4130A3 | fish | Macao | S-KF-K-SXT-KZ | O10 | + | - | - |
| 124 | 4130B3 | fish | Macao | S-KF-AMP-TE-K-SXT-KZ | O10 | + | - | - |
| 125 | 4131C2 | fish | Macao | S-KF-AMP-TE-C-K-AZM-KZ | O2 | + | - | - |
| 126 | 4134C1 | Deli meat | Macao | S-KF-AMP-TE-K-SXT-KZ | O10 | + | - | - |
| 127 | 4134C2 | Deli meat | Macao | AMP | O1 | + | - | - |
| 128 | 4135C3 | Deli meat | Macao | S-KF-AMP-K-CN-AZM-KZ | O12 | + | - | - |
| 129 | 4138C3 | Deli meat | Macao | S-KF | O1 | + | - | - |
| 130 | 4157A1 | fish | Hongkong | S-AMP | O2 | + | - | + |
| 131 | 4157A2 | fish | Hongkong | S-K-CN-KZ | O2 | + | - | - |
| 132 | 4157B3 | fish | Hongkong | S-AMP-TE-SXT-KZ | O2 | + | - | - |
| 133 | 4157B3 | fish | Hongkong | AMP-K-KZ | O2 | + | - | + |
| 134 | 4159B3 | Deli meat | Hongkong | S-K | O1 | + | + | - |
| 135 | 4161C2 | Deli meat | Hongkong | S-KZ | O2 | + | - | - |
| 136 | 4176A1 | fish | Hongkong | AMP-K-KZ | O1 | + | - | - |
| 137 | 4177B3 | fresh shrimp | Hongkong | S-KF-AMP-K-KZ | O2 | + | - | - |
| 138 | 4177C1 | fresh shrimp | Hongkong | S-KF-AMP-TE-K-CN-AZM-KZ | O2 | + | - | - |
| 139 | 4177C2 | fresh shrimp | Hongkong | S-AMP | O2 | + | - | - |
| 140 | 4178B1 | fish | Hongkong | AMP-K | O1 | + | - | - |
| 141 | 4179B2 | fish | Hongkong | S-KF-AMP-K | O2 | + | - | - |
| 142 | 4180A2 | fish | Hongkong | S-KF-AMP | O10 | + | - | - |
| 143 | 4180B1 | fish | Hongkong | S-KF-AMP-CN | O10 | + | - | - |
| 144 | 4206A1 | fish | Urumchi | S-KF-AMP-TE | O2 | + | - | - |
| 145 | 4227A2 | fresh shrimp | Urumchi | AMP-KZ | O10 | + | - | - |
| 146 | 4227B2 | fresh shrimp | Urumchi | S-KF-AMP-K-KZ | O10 | + | - | - |
| 147 | 4234C2 | Deli meat | Urumchi | S-KF-AMP-K-KZ | O2 | + | - | - |
| 148 | 545B1 | fresh shrimp | Changsha | S-KF-AMP-K | O1 | + | - | - |
| 149 | 549A2 | shelled shrimp | Changsha | NA-S-KF-AMP-CIP-K-CN-KZ | O1 | + | - | + |
| 150 | 549B3 | shelled shrimp | Changsha | S-KF-AMP-K-KZ | O1 | + | - | + |
| 151 | 554C1 | shelled shrimp | Changsha | S-KF-AMP-K-CN-KZ | O1 | + | - | - |
| 152 | 567A1 | fresh shrimp | Guiyang | S-KF-AMP-K-KZ | O1 | + | - | - |
| 153 | 567A3 | fresh shrimp | Guiyang | S-KF-AMP-KZ | O12 | + | - | - |
| 154 | 567B1 | fresh shrimp | Guiyang | NA-S-KF-K-KZ | O2 | + | - | - |
| 155 | 567C2 | fresh shrimp | Guiyang | S-KF-AMP | O2 | + | - | - |
| 156 | 567C3 | fresh shrimp | Guiyang | KF-AMP-KZ | O2 | + | - | - |
| 157 | 568A1 | fresh shrimp | Guiyang | S-KF-AMP-KZ | O2 | + | - | - |
| 158 | 568A2 | fresh shrimp | Guiyang | S-KF-AMP-KZ | O2 | + | - | - |
| 159 | 568A3 | fresh shrimp | Guiyang | S-KF-AMP-K-KZ | O12 | + | - | - |
| 160 | 569A1 | fresh shrimp | Guiyang | KF-AMP-KZ | O2 | + | - | - |
| 161 | 569A3 | fresh shrimp | Guiyang | S-KF | O12 | + | - | + |
| 162 | 569B2 | fresh shrimp | Guiyang | S-KF-AMP-KZ | O1 | + | - | - |
| 163 | 570B2 | fresh shrimp | Guiyang | S-KF-AMP | O1 | + | - | - |
| 164 | 578A1 | shelled shrimp | Guiyang | S-KF-AMP-KZ | O10 | + | - | + |
| 165 | 578C3 | shelled shrimp | Guiyang | S-KF-KZ | O2 | + | - | - |
| 166 | 593A3 | fresh shrimp | Sining | KF-TE-CN-KZ | O12 | + | - | + |
| 167 | 616A3 | fresh shrimp | Hohhot | S-AMP-K-CN-AZM | O10 | + | - | + |
| 168 | 616B1 | fresh shrimp | Hohhot | S-KF-AMP-K | O1 | + | - | + |
| 169 | 629B3 | fresh shrimp | Shenyang | S-KF-AMP-K-CN-AZM | O1 | + | - | - |
| 170 | 640A2 | fresh shrimp | Nanjing | KF-AMP-K | O12 | + | - | + |
| 171 | 640B2 | fresh shrimp | Nanjing | KF-AMP | O12 | + | - | + |
| 172 | 653A2 | fresh shrimp | Shijiazhuang | 0 | O12 | + | - | + |
| 173 | 709B1 | shelled shrimp | Macao | S-KF-AMP-AZM | O2 | + | + | + |
| 174 | 711C2 | fresh shrimp | Hongkong | AMP | O1 | + | - | - |
| 175 | 713A3 | fresh shrimp | Hongkong | KF-AMP | O1 | + | - | - |
| 176 | 714A1 | fresh shrimp | Hongkong | S-KF-AMP-K-CN-KZ | O2 | + | - | - |
| 177 | 714A3 | fresh shrimp | Hongkong | KF | O2 | + | + | - |
| 178 | 715A3 | shelled shrimp | Hongkong | AMP | O1 | + | - | - |
| 179 | 735A2 | fresh shrimp | Guiyang | S-AMP-K-KZ | uncertain | + | - | - |
| 180 | 735B1 | fresh shrimp | Guiyang | KF-AMP-KZ | uncertain | + | - | - |
| 181 | 735B3 | fresh shrimp | Guiyang | S-KF-AMP-KZ | O1 | + | - | - |
| 182 | 748A3 | fresh shrimp | Hangzhou | S-KF-AMP-KZ | O2 | + | - | - |
| 183 | 748B3 | fresh shrimp | Hangzhou | KF-AMP-K-KZ | O2 | + | - | - |
| 184 | 843A2 | fresh shrimp | Changchun | S-KF-AMP-TE-K-KZ | O1 | + | - | - |
| 185 | 855B1 | fresh shrimp | Macao | S-KF-AMP-K-KZ- | O2 | + | - | - |
| 186 | 855B2 | fresh shrimp | Macao | KF-AMP | O2 | + | - | - |
| 187 | 855C1 | fresh shrimp | Macao | S-KF | O2 | + | - | - |
| 188 | 855C3 | fresh shrimp | Macao | S-KF-AMP-TE-K-KZ | O10 | + | - | - |
| 189 | 857A1 | fresh shrimp | Macao | S-KF-AMP-K-CN-AZM-SXT-KZ | O2 | + | - | - |
| 190 | 857A2 | fresh shrimp | Macao | S-KF-K-CN-SXT-KZ | O1 | + | - | - |
| 191 | 857B3 | fresh shrimp | Macao | S-KF-AMP-TE-K-SXT-KZ | O2 | + | - | - |
| 192 | 857C3 | fresh shrimp | Macao | S-KF-AMP-TE-K-AZM-KZ | O2 | + | + | - |
| 193 | 858B1 | fresh shrimp | Macao | S-KF-AMP-CIP-CN-AZM-KZ | O2 | + | - | - |
| 194 | 858C2 | fresh shrimp | Macao | S-KF-AMP-CN-AZM-KZ | O2 | + | - | - |
| 195 | 860A1 | shelled shrimp | Macao | S-KF-AMP-TE-K-CN-KZ | O2 | + | - | - |
| 196 | 860B1 | shelled shrimp | Macao | S-KF-AMP-TE-K-KZ | O2 | + | - | - |
| 197 | 860B3 | shelled shrimp | Macao | S-KF-KZ | O2 | + | - | - |
| 198 | 867A1 | fresh shrimp | Hongkong | 0 | O2 | + | - | - |
| 199 | 877B3 | shelled shrimp | Hongkong | S-KF-AMP-KZ | O2 | + | - | - |
| 200 | 878A1 | shelled shrimp | Hongkong | AMP-KZ | O2 | + | - | - |
| 201 | 878A2 | shelled shrimp | Hongkong | KF | O2 | + | - | - |
| 202 | 878A3 | shelled shrimp | Hongkong | KF-CIP | O2 | + | - | - |
| 203 | ATCC17802 | ATCC | USA | AMP-KZ-KF | O1 | + | - | + |
| 204 | ATCC33847 | ATCC | USA | uncertain | O4 | + | + | - |
